# Chronic Exposure to Tributyltin Induces Brain Functional Damage in Juvenile Common Carp (*Cyprinus carpio*)

**DOI:** 10.1371/journal.pone.0123091

**Published:** 2015-04-16

**Authors:** Zhi-Hua Li, Ping Li, Ze-Chao Shi

**Affiliations:** 1 Key Laboratory of Freshwater Biodiversity Conservation (Ministry of Agriculture), Key Field Station for Fishery Resource and Environment in Upper-Middle Reaches of Yangtze River (Ministry of Agriculture), Yangtze River Fisheries Research Institute, Chinese Academy of Fishery Sciences, Wuhan 430223, China; 2 University of South Bohemia in Ceske Budejovice, Faculty of Fisheries and Protection of Waters, South Bohemian Research Center of Aquaculture and Biodiversity of Hydrocenoses, Research Institute of Fish Culture and Hydrobiology, Zátiší 728/II, 389 25 Vodňany, Czech Republic; Universidade Federal do Rio de Janeiro, BRAZIL

## Abstract

The aim of the present study was to investigate the effect of Tributyltin (TBT) on brain function and neurotoxicity of freshwater teleost. The effects of long-term exposure to TBT on antioxidant related indices (MDA, malondialdehyde; SOD, superoxide dismutase; CAT, catalase; GR, glutathione reductase; GPx, glutathione peroxidase), Na^+^-K^+^-ATPase and neurological parameters (AChE, acetylcholinesterase; MAO, monoamine oxidase; NO, nitric oxide) in the brain of common carp were evaluated. Fish were exposed to sublethal concentrations of TBT (75 ng/L, 0.75 μg/L and 7.5 μg/L) for 15, 30, and 60 days. Based on the results, a low level and short-term TBT-induced stress could not induce the notable responses of the fish brain, but long-term exposure (more than 15 days) to TBT could lead to obvious physiological-biochemical responses (based on the measured parameters). The results also strongly indicated that neurotoxicity of TBT to fish. Thus, the measured physiological responses in fish brain could provide useful information to better understand the mechanisms of TBT-induced bio-toxicity.

## Introduction

Tributyltin (TBT) is an organotin compound used primarily in anti-fouling paint applied on ships, boats and fishing nets [1]. As an endocrine disrupting chemical that causes severe reproductive effects in aquatic organisms, the use of TBT has been banned in most countries [2]. Based on the available data, the levels of TBT in aquatic environments of China range from below 0.5 ng/L (the detection limit) to hundreds of ng/L, as Tin [3]. Many literatures on the effects of TBT in biota has focused on reproductive toxicity. It has been demonstrated that TBT can induce imposex in female mollusks [4]. In fishes, it has been reported that TBT can affect sexual behaviour and reproduction [5], change the estrogen/androgen levels and inhibit gonad development [6]. Moreover, TBT-induced neurotoxicity, developmental toxicity and endocrine dysfunction have been reported in previous studies [7,8,9,10,11]. However, only a few studies have addressed the neurotoxicity of TBT especially in fish.

Oxidative stress is defined as an imbalance of oxidants and antioxidants in favor of the oxidants, potentially leading to cell damage [12]. Neurons are relatively sensitive to reactive oxygen species (ROS) and neurodegenerative disorders have been linked to damage caused by ROS [13,14]. As an organ in which homeostasis must be strictly maintained, brain tissue contains large amounts of polyunsaturated fatty acids, which are particularly vulnerable to free radical attacks [15]. Acetyl cholinesterase (AChE) and monoamine oxidase (MAO) play an important role in the central nervous system (CNS), including neurotransmitter release, synaptic plasticity, and the regulation of neuronal electrical activity [16]. Nitric oxide (NO) synthesized by nitric oxide synthase (NOS) has emerged as a key endogenous modulator of brain function [17]. Additional, Na^+^-K^+^-ATPase is a ubiquitous membrane-bound enzyme which concentrates in the membranes of nerve endings [18], and controls the ionic environment essential for neuronal activity in the central nervous system [19].

The mechanism of TBT-induced impairment of nervous system function, particularly in fish, remains unclear. In this study, juvenile common carp (*Cyprinus carpio*), as a widely used model in aquatic toxicology and an important economic fish, was exposed to TBT to determine its chronic effects on biochemical and physiological responses in fish brain. The objectives of the present study were to gain insight into the mechanisms underlying TBT-induced neural toxicity by assessing oxidative stress indices, Na^+^-K^+^-ATPase activity, and neurological parameters.

## Materials and methods

### Chemicals

TBT, 90%, was purchased from Sinopharm Chemical Reagent Co., Ltd (Beijing, China). Suitable amounts of this compound were directly weighed into a brown bottle and dissolved in 50 ml acetone (ACT)-water (1:1) to form a concentration level stability. This stock solution was sealed and stored at 4°C until used. Working standard solution (100 μg/ml) was freshly prepared by diluting the stock solution with deionized water before use.

### Fish

Juvenile common carp (9.65 ± 0.13 cm, 22 ± 1.8 g, 4 months after hatching) were obtained from a local hatchery (Jingzhou, China) and were raised in a flow-through system with dechlorinated tap water (pH 7.4 ± 0.2; hardness 42.5 ± 1.3 CaCO_3_/L) at a constant temperature (20 ± 1°C) with a photoperiod of 12:12 h (light:dark). Fish were acclimatized for 14 days before the beginning of the experiment and were fed commercial fish food (Tongwei, China). Waste and residue were removed daily and the test equipment and chambers (100 L) were cleaned once a week. The fish were fasted for 24 h prior to experimentation to avoid prandial effects during the assay. All procedures and animal handling were in accordance with the guidelines approved by the Chinese Association For Laboratory Animal Sciences. The study was approved by the animal ethics committee of the Institute of Yangtze River Fisheries Research Institute, Chinese Academy of Fishery Sciences.

### Exposure to TBT

A 100 L semi-static system was used, in which 20 juvenile common carp were randomly distributed to each of ten aquaria. The nominal concentrations of TBT used were 75 ng/L (E1 group, according to environmental concentration), 0.75 μg/L (E2 group, 1% 96 h-LC50), and 7.5 μg/L (E3 group, 10% 96 h-LC50). Based on the unpublished data of our studies, the 96 h-LC50 of TBT for common carp is 75 μg/L. TBT was dissolved in ACT with a final concentration less than 0.01%. Two other groups were used for comparisons: a control group exposed to clean freshwater and an ACT group exposed to the volume of ACT (v/v, 0.01%) used for the highest TBT concentration. Each experimental condition was duplicated. The fish were fed daily with commercial fish pellets at 1% total body weight at a fixed time and the extra food was removed. Eighty percent of the exposed solution was renewed each day after 2 h of feeding to maintain the appropriate concentration of TBT and ACT and to maintain water quality. The test equipment was cleaned every 7 days. The test fish were exposed to TBT for 15, 30 and 60 days. At the end of each exposure period, three randomly selected fish from each aquarium were sedated. The whole brain tissue was quickly removed on the ice, immediately frozen, and stored at -80°C until analysis.

To ensure agreement between nominal and actual compound concentrations in the aquaria, water samples were analyzed during the experimental period by LC-MS/MS. Water samples were collected from the test aquaria after 1 h and 24 h of renewing the test solutions. The mean concentration of TBT in the water samples was always within 20% of the intended concentration.

### Biochemical parameters measurement

Frozen samples for analysis of enzyme activities were defrosted and homogenized on ice with 10 volumes of cold Tris buffered saline (10 mM Tris–HCl, 0.1 mM EDTA-2Na, 10 mM sucrose, 0.8% NaCl, pH 7.4). The homogenate was centrifuged at 3000 rpm at 4°C for 10 min, and the supernatant was used to evaluate the enzyme activity of total superoxide dismutase (SOD; EC 1.15.1.1), catalase (CAT; EC 1.11.1.6),glutathione reductase (GR; EC 1.6.4.2), glutathione peroxidase (GPx; EC 1.11.1.9), Na-K-ATPase (EC 3.6.3.9), acetylcholinesterase (AChE; EC 3.1.1.7), monoamine oxidase (MAO: EC 1.4.3.4) and the content of malondialdehyde (MDA) and nitric oxide (NO). All of the biochemical parameters were measured using commercial kits (Nanjing Jiancheng Bioengineering Institute, Nanjing, China). Protein concentrations in the supernatants were determined using Bradford's procedure with bovine serum albumin as the standard (Bradford, 1976).

SOD (EC 1.15.1.1) activity was measured at 550 nm by the ferricytochrome C method using xanthine/xanthine oxidase as the source of superoxide radicals, and the activity was expressed as U/mg protein [20]. CAT (EC 1.11.1.6) activity was determined at 405 nm using a spectrophotometric assay of hydrogen peroxide, based on the formation of its stable complex with ammonium molybdate, and the activity was expressed as U/mg protein [21]. Glutathione reductase (EC 1.6.4.2) activity was determined spectrophotometrically, measuring NADPH oxidation at 340 nm [22]. GPx (EC 1.11.1.9) activity was measured using H_2_O_2_ as a substrate and was determined spectrophotometrically at 340 nm and 37°C, the activity was expressed in mU/mg protein [23]. MDA content, a biomarker for lipid peroxidation, was evaluated based on the 2-thiobarbituric acid (2,6-dihydroxypyrimidine-2-thiol; TBA) reactivity, and the results was expressed as nmol/mg protein [24]. Na^+^-K^+^-ATPase (EC 3.6.3.9) activity, expressed as μmol Pi liberated/mg protein/h in the brain, was measured by liberating PO_4_ from a hydrolysis reaction with ATPase, as previously described [25]. AChE (EC 3.1.1.7) activity in brain homogenates was determined with an AChE kit according to the method of Ellman et al. [26]. The activity of 1 U of AChE was defined as the number of hydrolyzed micromoles of acetylthiocholine iodide per min per microgram of protein. AChE activity in the brain homogenates was expressed as units per milligram of hippocampus protein (U/mg protein). The activity of MAO was determined using a detection kit, which assessed the production of benzyl aldehyde from the reaction of MAO and its specific substrate, aniline hydrochloride [27]. One unit (U) of MAO activity was defined as the amount that increased the absorbance by 0.01 at 37°C. Therefore, MAO activity was expressed as U/mg protein. NO content in brain homogenates was expressed as micromoles per milligram of hippocampus protein (μmol/mg protein) based on the method described by Stuehr et al. [28].

### Integrated biomarker response (IBR)

Biomarkers were combined into one general “stress index” termed IBR [29]. The result is directly dependent on the number of biomarkers (*n*) in the set and thus, IBR values were presented divided by *n* as suggested by Broeg and Lehtonen [30]. The results of the data standardization procedure needed for IBR calculation were presented in site star plots.

### Data statistical assays

All values were expressed as the mean ± SD and analyzed by SPSS for Win 13.0 software. Analyses of variance (one-way and two-way ANOVA), followed by a Tukey HSD test when significant differences were found, was performed to determine the effect of TBT concentration and exposure time on each parameters. In addition, principal component analysis (PCA) was used to define the most important parameters, which could be used as key factors for individual variations using Statistic 6.0.

## Results

### Oxidative stress and antioxidant responses

To verify the presence of oxidative imbalance induced by TBT, the level of MDA (as indicated by tissue ROS level) was measured in all groups (Table 1). Although an increase of oxidative stress indices was observed, there was no notable induction (*p*>0.05) in any group after 15 days of TBT exposure. Compared with the control group, a significant increase (*p*<0.05) in MDA level was observed in the E2 and E3 groups after 30 days of exposure.

**Table 1:** Effect of TBT on antioxidant parameters in the brain of common carp (*Cyprinus carpio*).

Indices	Exposure time	Test groups				
		Control	ACT	E1	E2	E3
MDA	15 days	16.28 ± 1.32	14.75 ± 0.82	16.65 ± 1.62	16.98 ± 2.07	18.89 ± 2.26
	30 days	15.19 ± 1.94	16.05 ± 1.29	18.28 ± 1.95	24.54 ± 3.26*	29.21 ± 1.89*
	60 days	17.57 ± 2.69	18.39 ± 1.33	21.35 ± 2.83	35.16 ± 2.09*	38.07 ± 4.18**
SOD	15 days	31.27 ± 2.58	30.01 ± 4.06	34.54 ± 4.15	36.26 ± 4.17	35.17 ± 4.75
	30 days	32.15 ± 3.15	36.24 ± 4.23	38.58 ± 3.29	36.41 ± 5.02	24.17 ± 3.56*
	60 days	31.69 ± 2.99	33.18 ± 4.41	26.82 ± 2.16	15.44 ± 1.04*	10.30 ± 1.82**
CAT	15 days	25.15 ± 3.59	26.04 ± 1.81	26.28 ± 3.01	27.42 ± 2.25	29.47 ± 3.97*
	30 days	24.27 ± 3.14	25.29 ± 2.72	27.77 ± 3.82	20.42 ± 1.94	15.85 ± 1.49*
	60 days	25.70 ± 2.85	24.16 ± 1.57	26.25 ± 2.67	17.19 ± 1.37*	11.44 ± 1.85**
GR	15 days	22.15 ± 2.12	21.82 ± 1.58	22.35 ± 2.14	20.24 ± 1.69	19.91 ± 1.12
	30 days	23.89 ± 1.93	20.46 ± 1.72	19.61 ± 1.57	20.45 ± 1.92	17.72 ± 1.24*
	60 days	22.27 ± 2.08	21.53 ± 1.34	19.29 ± 2.05	16.28 ± 1.17*	14.19 ± 1.05*
GPx	15 days	65.28 ± 11.02	63.14 ± 12.28	72.49 ± 14.59	82.31 ± 14.41*	69.42 ± 11.22
	30 days	62.12 ± 9.34	66.25 ± 10.22	69.27 ± 11.67	61.19 ± 13.28	35.22 ± 9.85**
	60 days	64.27 ± 10.19	61.19 ± 14.06	50.26 ± 10.38	41.37 ± 7.72*	24.06 ± 6.28**

Notes: MDA, malondialdehyde, nmol/mg protein; SOD, superoxide dismutase, U/mg protein; CAT, catalase, U/mg protein; GR, glutathione reductase, mU/mg protein; GPx, glutathione peroxidase, mU/mg protein. ACT- acetone, E1-75 ng/L, E2-0.75 μg/L, E3-7.5 μg/L. Data are means ± S.D., *n* = 6. Significant differences compared with control values are presented by:

* *p*<0.05

** *p*<0.01

Table 1 shows the effects of long-term exposure to TBT on antioxidant parameters in fish brain. After 15 days, SOD activities increased slightly in all groups, but significantly decreased (*p*<0.05) in groups with higher TBT concentration after 30 days (E3 group after 30 days; E2 and E3 groups after 60 days). For CAT activity, there was an increasing trend in all groups after 15 days of exposure, and it was greater increased (*p*<0.05) in the E3 group. However, CAT activities in E2 or/and E3 group were inhibited significantly (*p*<0.05) after 30 days (E3 group after 30 days; E2 and E3 groups after 60 days). After the first 15 days of exposure, GR activities of all groups decreased slightly but not significantly (*p*>0.05). After 30 days, GR activity was significantly inhibited (*p*<0.05) in the E3 group. At the end of experiment, there was an obviously decreasing trend in GR activities in all groups, which reached significance (*p*<0.05) in the E2 and E3 groups. There were greater increases (*p*<0.05) in GPx activity in the E2 group after 15 days. Glutathione peroxidase activities were obviously inhibited (*p*<0.05) in the E2 and/or E3 group after 30 days (E3 group after 30 days; E2 and E3 groups after 60 days).

### Neurological parameters

Table 2 shows the effects of long-term exposure to TBT on neurological parameters in the brain of common carp. Compared with the control, significantly lower (*p*<0.05) AChE activity was observed in the E3 group after 15 days of exposure, in the E2 group after 30 days and in the E1 group after 60 days. After 30 days of exposure, there was an obviously decrease (*p*<0.05) in MAO activity in the E3 group compared with the controls. However, after 60 days of treatment, MAO activity in E2 and E3 groups were significantly inhibited (*p*<0.05) compared with the control. As for NO, there was a decreasing trend in TBT treated groups after 15 days. With prolonged exposure (30 days), the NO level was significantly inhibited (*p*<0.05) in the E2 and E3 groups.

**Table 2:** Effects of TBT on neurological parameters in the brain of common carp (*Cyprinus carpio*).

Indices	Exposure time	Test groups				
		Control	ACT	E1	E2	E3
AChE	15 days	5.22 ± 1.52	5.62 ± 0.92	5.51 ± 1.62	4.92 ± 1.07	3.51 ± 1.26*
	30 days	5.30 ± 1.25	5.14 ±0.89	4.78 ± 1.15	4.44 ± 1.24*	2.55 ± 0.81*
	60 days	5.19 ± 0.91	5.27 ± 1.03	3.37 ± 0.89*	1.62 ± 0.69**	1.27 ± 1.15**
MAO	15 days	11.16 ± 1.58	10.21 ± 1.16	10.54 ± 1.15	9.76 ± 0.97	9.07 ± 1.25
	30 days	12.27 ± 1.05	10.13 ± 1.57	8.52 ± 0.93	8.47 ± 1.02	6.16 ± 0.59*
	60 days	11.58 ± 1.33	9.47 ± 1.43	8.47 ± 1.36	6.54 ± 0.84*	5.34 ± 0.82**
NO	15 days	1.27 ± 0.25	1.20 ± 0.19	1.28 ± 0.18	1.05 ± 0.22	0.98 ± 0.17
	30 days	1.19 ± 0.14	1.06 ± 0.16	1.05 ± 0.12	0.82 ± 0.14*	0.76 ± 0.09*
	60 days	1.14 ± 0.20	1.13 ± 0.17	0.89 ± 0.19	0.68 ± 0.10*	0.44 ± 0.05**

Notes: AChE, acetylcholinesterase, U/mg protein; MAO, monoamine oxidase, U/mg protein; NO, nitric oxide, mmol/mg protein. ACT- acetone, E1-75 ng/L, E2-0.75 μg/L, E3-7.5 μg/L. Data are means ± S.D., *n* = 6. Significant differences compared with control values are presented by:

* *p*<0.05

** *p*<0.01

### Na^+^-K^+^-ATPase

**Fig 1:**
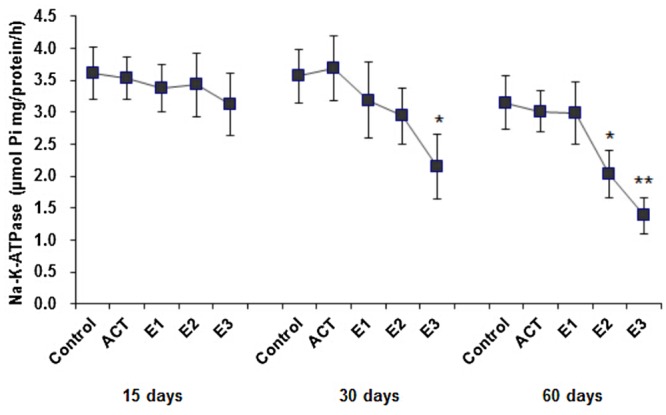
Effect of TBT exposure on Na^+^-K^+^-ATPase enzyme activity in the brain of common carp (*Cyprinus carpio*). Data are presented as the mean ± S.D., *n* = 6 for each data point. ACT- acetone, E1-75 ng/L, E2-0.75 μg/L, E3-7.5 μg/L. Significant differences compared with control values are indicated by * *p*<0.05, ** *p*<0.01.

Na^+^-K^+^-ATPase activities in the brains of fish exposed to TBT for 15, 30 and 60 days re shown in Fig 1. Na^+^-K^+^-ATPase activity was diminished in both a time- and concentration-dependent manner. Significant depletion (*p*<0.05) in Na^+^-K^+^-ATPase activities occurred in the E3 group after 30 days, and in the E2 group after 60 days.

### Statistical analysis

**Fig 2:**
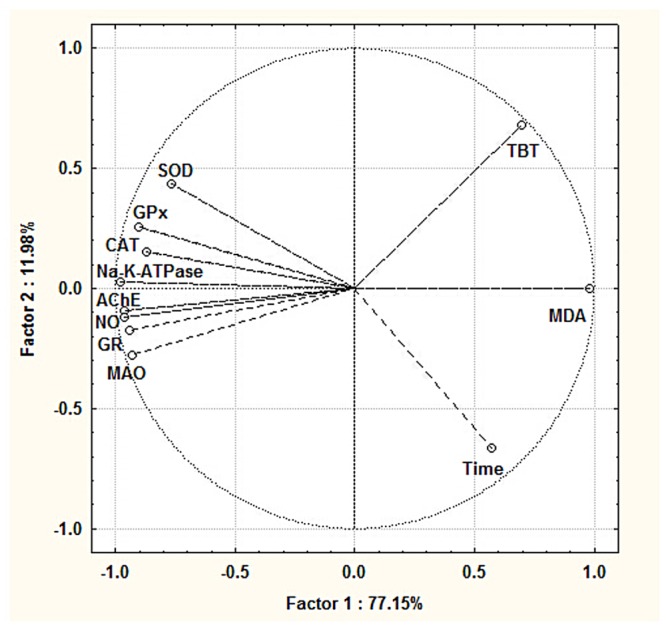
Ordination diagram of PCA of TBT concentrations, exposure time and all parameters measured in the fish brains after chronic exposure to TBT.

Based on the bilinear decomposition of the original data, the PCA method was used to transform the multivariate data into a new data set, in which the new variables are orthonormal and explain maximum. In the present study, a data matrix was constructed with 9 analyzed biomarkers as independent variables and 90 sampled individuals as group variables. TBT concentration, exposure time and all of the parameters measured in the present study were distinguished on the ordination plots corresponding to the first (77.15%) and second (11.98%) principle components (Fig 2), which showed the correlations of all the biomarkers. Furthermore, the observed correlations between the TBT concentrations, exposure time and the parameters were confirmed and quantified by Spearman’s test (Table 3). Moreover, Two-way ANOVA, using all parameters measured as the dependent variables, and TBT concentrations and exposure time as fixed factors, revealed the notable variation in each parameter with different experimental conditions (Table 4).

**Table 3:** Correlation coefficients among the biochemical parameters measured in the brain of common carp (*Cyprinus carpio*) after long-term exposure to TBT.

	Time	TBT	MDA	SOD	CAT	GR	GPx	AChE	MAO	NO
MDA	0.54	0.68								
SOD	-0.61	-0.25	-0.76							
CAT	-0.46	-0.46	-0.90	0.68						
GR	-0.45	-0.75	-0.90	0.68	0.73					
GPx	-0.60	-0.42	-0.87	0.76	0.90	0.76				
AChE	-0.49	-0.74	-0.94	0.73	0.76	0.93	0.84			
MAO	-0.39	-0.83	-0.90	0.55	0.74	0.95	0.77	0.89		
NO	-0.51	-0.76	-0.94	0.66	0.80	0.94	0.81	0.94	0.91	
Na^+^-K^+^-ATPase	-0.59	-0.68	-0.97	0.74	0.87	0.89	0.89	0.92	0.90	0.91

Notes: TBT, tributyltin; MDA, malondialdehyde; SOD, superoxide dismutase; CAT, catalase; GR, glutathione reductase; GPx, glutathione peroxidase; AChE, acetylcholinesterase; MAO, monoamine oxidase; NO, nitric oxide.

**Table 4:** Two-way ANOVA of variance for the effects of TBT concentrations (TBT) and exposure time (Time) on parameters measured in the brain of common carp (*Cyprinus carpio*) after long-term exposure to TBT.

Indices	Experimental conditions		
	Time	TBT	Time*TBT
MDA	**<0.001**	**<0.001**	**<0.001**
SOD	**<0.001**	**0.012**	**<0.001**
CAT	**0.007**	**0.002**	**<0.001**
GR	**0.005**	**<0.001**	**<0.001**
GPx	**<0.001**	**0.003**	**<0.001**
AChE	**0.004**	**<0.001**	**<0.001**
MAO	**0.013**	**<0.001**	**<0.001**
NO	**<0.001**	**<0.001**	**<0.001**
Na^+^-K^+^-ATPase	**<0.001**	**<0.001**	**<0.001**

Notes: Data were expressed by significant value.

Based on the results (Table 4), all the measured parameters were affected by the TBT concentration, exposure time and the interaction between the two factors. But it is obvious that some parameters were more sensitive to the exposure time, including MDA, SOD, GPx, NO and Na^+^-K^+^-ATPase, however the parameters MDA, GR, AChE, MAO, NO and Na^+^-K^+^-ATPase were more sensitive to the TBT concentrations.

**Fig 3:**
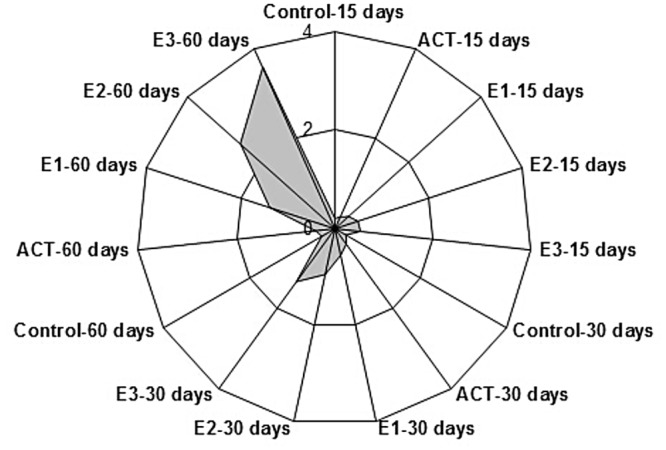
Integrated biomarker response (IBR) of all parameters measured in the fish brain after chronic exposure to TBT. ACT- acetone, E1-75 ng/L, E2-0.75 μg/L, E3-7.5 μg/L.

IBR index showed E3-60 days as the most affected group. IBR values ranged from 0.21 in the Control-15 days up to 3.59 in E3-60 days (Fig 3). According to this index, the rank of the most affected group was: E3-60 days > E2-60 days > E1-60 days > E3-30 days > E2-30 days > E1-30 days > E3-15 days > E2-15 days > ACT-60 days > ACT-30 days > E1-15 days > Control-60 days > Control-30 days > ACT-15 days > Control-15 days.

## Discussion

### Oxidative stress and antioxidant responses

Oxidative stress, the final manifestation of a multi-step pathway, results in an imbalance between pro-oxidant and antioxidant defense mechanisms due to the depletion of antioxidants, or the excessive accumulation of ROS, or both, which leads to damage [31]. It has been demonstrated that exposure to contaminants including TBT could produce ROS which cause various organ lesions [32,33,34].

MDA, as lipid peroxidation indicator, was used to evaluate the oxidative stress for aquatic animals [35]. In the present study, the significantly increasing MDA level indicated the generation of the serious oxidative stress in fish exposed to TBT.

Due to the inhibitory effects on oxyradical formation, the SOD-CAT system provides the first line of defense against oxygen toxicity [36]. In the present study, the activity of SOD and CAT of in the TBT treated groups increased slightly after 15 days of exposure compared to that of the control group, especially in the increase in CAT activity at the highest concentration of TBT (Table 1). Increases in the activity of these enzymes are most likely a response to toxicant stress, and serve to neutralize the impact of increased ROS generation [37]. However, we also found that SOD and CAT activities in fish brains were strongly inhibited with prolonged duration of exposure, which could be due to the flux of superoxide radicals, resulting in an increase in cellular H_2_O_2_ [38]. GR plays an important role in cellular antioxidant protection and adjustment processes of metabolic pathways, and GPx catalyzes the reduction of H_2_O_2_ and lipid peroxides [39]. Together they constitute an important line of defense against oxidative stress-induced by xenobiotics. In this study, GR and GPx were inhibited at higher TBT concentrations, suggesting that the TBT-induced accumulation of ROS has interfered with the antioxidant enzymes in the brains of exposed fish. Some reports indicated that an increase in the cellular level of oxygen free radicals following TBT-mitochondria interactions may be the cause of the TBT-induced functional damage in fish model [40,41]. So together with previous results [42,43,44,45], our study indicated that the possible reason of TBT-induced oxidative stress maybe TBT inhibit oxidative phosphorilation and alter mitochondrial structure and function.

Generally, oxidative stress refers to imbalance cellular status between the ROS level and the cellular antioxidant defense system. Approximately 0.1% of all oxygen entering the mitochondrial electron transport chain is released as ROS, which can disrupt intracellular redox status and result in homeostasis disorder [46]. In this work, a significant correlation was observed between the antioxidant parameters to TBT exposure, which suggested that oxidative stress was induced by TBT. Combining previous results [34] with the findings of this study, suggested that oxidative damage is one of the critical toxic mechanisms of TBT. Because the special physiological characteristics with more mitochondrial structure in fish brain, the possible mechanisms of TBT-induced oxidative stress maybe a higher oxygen consumption rate to increase metabolic rate to detoxify TBT toxicity eventually led to higher ROS production, which need more further studies.

### Neurological parameters

AChE plays a crucial role in synaptic transmission at cholinergic synapses by controlling the action of acetylcholine [47]. MAO also plays a vital role in the metabolism of neurotransmitters such as dopamine, norepinephrine, epinephrine, and serotonin [48]. Dysfunctions of AChE and MAO have been implicated in a variety of neuropsychiatric disorders [16]. Similar to the findings of other studies, we deduced that the inhibition of AChE and MAO activities under TBT stress may occur due to the altered affinity for free-SH groups and consequential inhibition of their function [49].

NO plays an important role in cell signaling, neurotransmission, cell-protection and regulatory effects in various cells at a physiological concentrations [50], some tests had been carried out in Atlantic salmon [17]. Therefore, alterations in NO production may be a causal factor in the development of neurotoxicity [51]. On the other hand, high concentrations of NO are toxic and interact with the superoxide anion to produce the highly toxic peroxynitrite anion [52]. In the present study, we found that TBT decreased NO production in the brains of exposed fish (Table 2). Moreover, as previously described, we observed that TBT markedly increased ROS levels in fish brains. The over-production of ROS can decrease NO levels in the brain because NO reacts rapidly with O_2_•^−^ to produce the peroxynitrite anion (ONOO-), which protonates at relevant pH to form peroxynitrous acid (ONOOH) [16]. Both ONOO- and ONOOH can cause nitrosative stress, which may lead to nitrosylation reactions that can alter the structure of proteins and so inhibit their normal function [50].

Previous findings support the view that ROS contribute to the induction of neurodegenerative disorders [53]. Zhang et al reported that the TBT exposure could injured the neurological function in fish brain [34], associated with oxidative stress. Based on Table 3, the obvious negative correlation between neurological parameters and oxidative stress (MDA content) in fish brain after TBT exposure could provide another evident.

### Na^+^-K^+^-ATPase

Ion-dependent ATPases comprise a group of enzymes that play an important role in intracellular functions and are considered to be a sensitive indicator of toxicity [25,54]. These enzymes, especially Na^+^-K^+^-ATPase, are essential for the generation and maintenance of Na^+^ and K^+^ gradients between the intracellular and extracellular milieus, a prerequisite for basic cellular homeostasis and for functioning of specialized tissues, such as the nervous system [34]. The inhibition of this enzyme activity produces membrane depolarization, leading to the suppression of neuronal and excitatory transmission [19]. It is reported that organotins such as tributyltinoxide, trimethyltin, triethyltin and dibenzyltin suppress Na^+^/K^+^-ATPase activity in the brain [55,56]. Published studies showed that organotins affected the Na^+^/K^+^-ATPase activity and osmoregulation in European flounder, *Platichthys flesus* [57], through adjusting the Na^+^/K^+^ flux. In our study, there is a very obvious negative correlation between Na^+^/K^+^-ATPase activity and MDA, neurological parameters. The possible reasons may be related to the special physiological characteristics in fish brain. Brain tissue, as the center of the nervous system in all vertebrate, contains large amounts of polyunsaturated fatty acids, which are particularly vulnerable to free radical attacks [15]. Zhang et al [34] also reported that TBT exposure caused the brain damage in False kelpfish, *Sebastiscus marmoratus*, associated with increasing of ROS stress and decreasing of Na^+^/K^+^-ATPase activity. Similar to these findings, in the present study we observed that the TBT-induced inhibition of Na^+^-K^+^-ATPase in the fish brain after long-term exposure most likely disturbs the Na-K pump, which could then be responsible for the TBT neurotoxicity. [58].

#### IBR analysis

In order to compare the overall stress of different treatments on fish liver, the IBR index was applied. Recently, this index has been used to provide a general description of “health status” of organisms by combining biomarker signals, from which the rank of chemical stress could be ordered [59,60,61]. In our study, some large IBR values were possessed by high-dose TBT groups for the same exposure time, implying the more serious stress with increasing TBT concentration. This tendency may be explained by more ROS production and causing more severe injuries to antioxidant system. Similar results were also found in Oncorhynchus mykiss [61] and Carassius auratus [62] exposed to verapamil and hexabromobenzene at various ranges of concentrations, respectively. IBR results suggest that E3-60days was the most affected group, which is consistent with the analysis for all measured parameters.

In summary, the present study demonstrated changes in oxidative stress indices, antioxidant defense system, neurological parameters and Na^+^/K^+^-ATPase activity in the brain of common carp after long-term exposure to TBT. Based on our results, there is an “adaptive stage” (15 days), in which all physiological indices remained at control values due to protective responses. With prolonged exposure, TBT-induced stress led to functional damage in the brains of the fish, by accumulation of oxidative substances, inhibition of antioxidant defences and inhibition of the neurological system and Na^+^-K^+^-ATPase activity. According to the results of this study, the measured parameters could be used as potential biomarkers and could provide useful information for evaluating the toxicological effects of TBT on fish brains, but additional detailed laboratory studies are required to elucidate the molecular mechanisms of TBT neuro-toxicity in other organisms.

## References

[1] Mortensen AS, Arukwe A (2007) Modulation of xenobiotic biotransformation system and hormonal responses in Atlantic salmon (*Salmo salar*) after exposure to tributyltin (TBT). Comp Biochem Physiol C-Toxicol Pharmacol 145: 431–441. 17344101 10.1016/j.cbpc.2007.01.013

[2] Antizar-Ladislao B (2008) Environmental levels, toxicity and human exposure to tributyltin (TBT)-contaminated marine environment. a review. Environ Int 34: 292–308. 17959247 10.1016/j.envint.2007.09.005

[3] Gao JM, Hu JY, Zhen H, Yang M, Li BZ (2006) Organotin compounds in the Three Gorges Reservoir region of the Yangtze River. Bull Environ Contam Toxicol 76: 155–162. 16404674 10.1007/s00128-005-0902-x

[4] Morcillo Y, Porte C (2000) Evidence of endocrine disruption in clams—*Ruditapes decussata*—transplanted to a tributyltin-polluted environment. Environ Pollut 107: 47–52. 15093007 10.1016/s0269-7491(99)00133-5

[5] Nakayama K, Oshima Y, Yamaguchi T, Tsuruda Y, Kang IJ, Kobayashi M, et al (2004) Fertilization success and sexual behavior in male medaka, *Oryzias latipes*, exposed to tributyltin. Chemosphere 55: 1331–1337. 15081776 10.1016/j.chemosphere.2003.11.050

[6] Zhang J, Zuo Z, Chen Y, Zhao Y, Hu S, Wang C (2007) Effect of tributyltin on the development of ovary in female cuvier (*Sebastiscus marmoratus*). Aquat Toxicol 83: 174–179. 17512063 10.1016/j.aquatox.2007.03.018

[7] St-Jean SD, Pelletier E, Courtenay SC (2002) Very low levels of waterborne butyltins modulate hemocyte function in the blue mussel *Mytilus edulis* . Mar Ecol Prog Ser 236: 155–161.

[8] Horiguchi T (2006) Masculinization of female gastropod mollusks induced by organotin compounds, focusing on mechanism of actions of tributyltin and triphenyltin for development of imposex. Environ Sci 13: 77–87. 16788559

[9] Porte C, Janer G, Lorusso LC, Ortiz-Zarragoitia M, Cajaraville MP, Fossi MC, et al (2006) Endocrine disruptors in marine organisms: approaches and perspectives. Comp Biochem Physiol C-Toxicol Pharmacol 143: 303–315. 16723279 10.1016/j.cbpc.2006.03.004

[10] Mitra S, Siddiqui WA, Khandelwal S (2014) Differential susceptibility of brain regions to tributyltin chloride toxicity. Environ Toxicol 10.1002/tox.22009 24895210

[11] Park K, Kim R, Park JJ, Shin HC, Lee JS, Cho HS, et al (2012) Ecotoxicological evaluation of tributyltin toxicity to the equilateral venus clam, *Gomphina veneriformis* (Bivalvia: Veneridae). Fish Shellfish Immunol 32: 426–433. 10.1016/j.fsi.2011.11.031 22182740

[12] Lushchak VI (2011) Environmentally induced oxidative stress in aquatic animals. Aquat Toxicol 101: 13–30. 10.1016/j.aquatox.2010.10.006 21074869

[13] Li ZH, Zlabek V, Grabic R, Li P, Machova J, Velisek J, et al (2010) Effects of exposure to sublethal propiconazole on the antioxidant defense system and Na^+^-K^+^-ATPase activity in brain of rainbow trout, *Oncorhynchus mykiss* . Aquat Toxicol 98: 297–303. 10.1016/j.aquatox.2010.02.017 20363517

[14] Li ZH, Zlabek V, Velisek J, Grabic R, Machova J, Randak T (2010) Modulation of antioxidant defence system in brain of rainbow trout (*Oncorhynchus mykiss*) after chronic carbamazepine treatment. Comp Biochem Physiol C 151: 137–141. 10.1016/j.cbpc.2009.09.006 19778632

[15] Sahin E, Gumuslu S (2004) Alterations in brain antioxidant status, protein oxidation and lipid peroxidation in response to different stress models. Behav Brain Res 155: 241–248. 15364483 10.1016/j.bbr.2004.04.022

[16] Liu CM, Zheng GH, Ming QL, Sun JM, Cheng C (2013) Protective effect of puerarin on lead-induced mouse cognitive impairment via altering activities of acetyl cholinesterase, monoamine oxidase and nitric oxide synthase. Environ Toxicol Pharmacol 35: 502–510. 10.1016/j.etap.2013.02.009 23501611

[17] Holmqvist B, Ekstrom P (1997) Subcellular localization of neuronal nitric oxide synthase in the brain of a teleost; an immunoelectron and confocal microscopical study. Brain Res 745: 67–82. 9037395 10.1016/s0006-8993(96)01128-6

[18] Rodriguez de Lores A, Alberici M, De Robertis E (1967) Ultrastructural and enzymic studies of cholinergic and non-cholinergic synaptic membranes isolated from brain cortex. J Neurochem 14: 215–225. 4289877 10.1111/j.1471-4159.1967.tb05897.x

[19] Balestrino M, Young J, Aitken P (1999) Block of (Na+,K+)ATPase with ouabain induces spreading depression-like depolarization in hippocampal slices. Brain Res 838: 37–44. 10446314 10.1016/s0006-8993(99)01674-1

[20] McCord JM, Fridovich I (1969) Superoxide dismutase. An enzymic function for erythrocuprein (hemocuprein). J Biol Chem 244: 6049–6055. 5389100

[21] Goth L (1991) A simple method for determination of serum catalase activity and revision of reference range. Clin Chim Acta 196: 143–151. 2029780 10.1016/0009-8981(91)90067-m

[22] Carlberg I, Mannervik B (1975) Purification and characterization of flavoenzyme glutathione reductase from rat liver. J Biol Chem 250: 5475–5480. 237922

[23] Lawrence RA, Burk RF (1976) Glutathione peroxidase activity in selenium deficient rat liver. Biochem Biophys Res Commun 71: 952–958. 971321 10.1016/0006-291x(76)90747-6

[24] Jain SK, McVie R, Duett J, Herbst JJ (1989) Erythrocyte membrane lipid peroxidation and glycosylated hemoglobin in diabetes. Diabetes 38: 1539–1543. 2583378 10.2337/diab.38.12.1539

[25] Agrahari S, Gopal K (2008) Inhibition of Na^+^-K^+^-ATPase in different tissues of freshwater fish *Channa punctatus* (Bloch) exposed to monocrotophos. Pest Biochem Physiol 92: 57–60.

[26] Ellman GL, Courtney KD, Andres V Jr., Feather-Stone RM (1961) A new and rapid colorimetric determination of acetylcholinesterase activity. Biochem Pharmacol 7: 88–95. 13726518 10.1016/0006-2952(61)90145-9

[27] Tabakoff B, Alivasatos SG (1972) Modified method for spectrophotometric determination of monoamine oxidase activity. Anal Chem 44: 427–428. 5059094 10.1021/ac60310a040

[28] Stuehr DJ, Kwon NS, Gross SS, Thiel BA, Levi R, Nathan CF (1989) Synthesis of nitrogen oxides from L-arginine by macrophage cytosol: requirement for inducible and constitutive components. Biochem Biophys Res Commun 161: 420–426. 2735902 10.1016/0006-291x(89)92615-6

[29] Beliaeff B, Burgeot T (2002) Integrated biomarker response: A useful tool for ecological risk assessment. Environ Toxicol Chem 21: 1316–1322. 12069320

[30] Broeg K, Lehtonen KK (2006) Indices for the assessment of environmental pollution of the Baltic Sea coasts: Integrated assessment of a multi-biomarker approach. Mar Pollut Bull 53: 508–522. 16737720 10.1016/j.marpolbul.2006.02.004

[31] Kavitha P, Rao JV (2009) Sub-lethal effects of profenofos on tissue-specific antioxidative responses in a Euryhyaline fish, *Oreochromis mossambicus* . Ecotoxicol Environ Saf 72: 1727–1733. 10.1016/j.ecoenv.2009.05.010 19501401

[32] Livingstone DR (2001) Contaminant-stimulated reactive oxygen species production and oxidative damage in aquatic organisms. Mar Pollut Bull 42: 656–666. 11525283 10.1016/s0025-326x(01)00060-1

[33] Casalino E, Calzaretti G, Sblano C, Landriscina C (2002) Molecular inhibitory mechanisms of antioxidant enzymes in rat liver and kidney by cadmium. Toxicology 179: 37–50. 12204541 10.1016/s0300-483x(02)00245-7

[34] Zhang J, Zuo Z, Chen R, Chen Y, Wang C (2008) Tributyltin exposure causes brain damage in *Sebastiscus marmoratus* . Chemosphere 73: 337–343. 10.1016/j.chemosphere.2008.05.072 18644613

[35] Rajeshkumar S, Mini J, Munuswamy N (2013) Effects of heavy metals on antioxidants and expression of HSP70 in different tissues of Milk fish (*Chanos chanos*) of Kaattuppalli Island, Chennai, India. Ecotox Environ Safe 98: 8–18. 10.1016/j.ecoenv.2013.07.029 24021871

[36] Pandey S, Parvez S, Sayeed I, Haque R, Bin-Hafeez B, Raisuddin S (2003) Biomarkers of oxidative stress: a comparative study of river Yamuna fish *Wallago attu* (Bl. & Schn.). Sci Total Environ 309: 105–115. 12798096 10.1016/S0048-9697(03)00006-8

[37] John S, Kale M, Rathore N, Bhatnagar D (2001) Protective effect of vitamin E in dimethoate and malathion induced oxidative stress in rat erythrocytes. Journal of Nutritional Biochemistry 12: 500–504. 11834209 10.1016/s0955-2863(01)00160-7

[38] Zhang X, Yang F, Zhang X, Xu Y, Liao T, Song S, et al (2008) Induction of hepatic enzymes and oxidative stress in Chinese rare minnow (*Gobiocypris rarus*) exposed to waterborne hexabromocyclododecane (HBCDD). Aquat Toxicol 86: 4–11. 18022707 10.1016/j.aquatox.2007.07.002

[39] Li ZH, Zlabek V, Grabic R, Li P, Randak T (2010) Modulation of glutathione-related antioxidant defense system of fish chronically treated by the fungicide propiconazole. Comp Biochem Physiol C 152: 392–398. 10.1016/j.cbpc.2010.06.006 20601116

[40] Tiano L, Fedeli D, Santoni G, Davies I, Falcioni G (2003) Effect of tributyltin on trout blood cells: changes in mitochondrial morphology and functionality. Biochim Biophys Acta-Mol Cell Res 1640: 105–112. 12729919 10.1016/s0167-4889(03)00025-9

[41] Tiano L, Fedeli D, Moretti M, Falcioni G (2001) DNA damage induced by organotins on trout-nucleated erythrocytes. Appl Organomet Chem 15: 575–580.

[42] Aldridge WN, Street BW (1970) Oxidative phosphorylation. The specific binding of trimethyltin and triethyltin to rat liver mitochondria. Biochem J 118: 171–179. 5472149 10.1042/bj1180171 PMC1179094

[43] Gennari A, Viviani B, Galli CL, Marinovich M, Pieters R, Corsini E (2000) Organotins induce apoptosis by disturbance of [Ca(2+)](i) and mitochondrial activity, causing oxidative stress and activation of caspases in rat thymocytes. Toxicol Appl Pharmacol 169: 185–190. 11097871 10.1006/taap.2000.9076

[44] Mitra S, Siddiqui WA, Khandelwal S (2014) Early cellular responses against tributyltin chloride exposure in primary cultures derived from various brain regions. Environ Toxicol Pharmacol 37: 1048–1059. 10.1016/j.etap.2014.03.020 24762416

[45] Nesci S, Ventrella V, Trombetti F, Pirini M, Pagliarani A (2011) Tributyltin (TBT) and mitochondrial respiration in mussel digestive gland. Toxicol Vitro 25: 951–959. 10.1016/j.tiv.2011.03.004 21396439

[46] Zhou J, Zhu XS, Cai ZH (2010) Tributyltin toxicity in abalone (*Haliotis diversicolor supertexta*) assessed by antioxidant enzyme activity, metabolic response, and histopathology. J Hazard Mater 183: 428–433. 10.1016/j.jhazmat.2010.07.042 20709453

[47] Basha DC, Rani MU, Devi CB, Kumar MR, Reddy GR (2012) Perinatal lead exposure alters postnatal cholinergic and aminergic system in rat brain: reversal effect of calcium co-administration. Int J Dev Neurosci 30: 343–350. 10.1016/j.ijdevneu.2012.01.004 22326442

[48] Devi CB, Reddy GH, Prasanthi RP, Chetty CS, Reddy GR (2005) Developmental lead exposure alters mitochondrial monoamine oxidase and synaptosomal catecholamine levels in rat brain. Int J Dev Neurosci 23: 375–381. 15927761 10.1016/j.ijdevneu.2004.11.003

[49] Reddy GR, Devi BC, Chetty CS (2007) Developmental lead neurotoxicity: alterations in brain cholinergic system. Neurotoxicology 28: 402–407. 16678265 10.1016/j.neuro.2006.03.018

[50] Nava-Ruiz C, Alcaraz-Zubeldia M, Mendez-Armenta M, Vergara P, Diaz-Ruiz A, Rios C (2010) Nitric oxide synthase immunolocalization and expression in the rat hippocampus after sub-acute lead acetate exposure in rats. Exp Toxicol Pathol 62: 311–316. 10.1016/j.etp.2009.04.006 19524414

[51] Kim S, Hyun J, Kim H, Kim Y, Kim E, Jang J, et al (2011) Effects of lead exposure on nitric oxide-associated gene expression in the olfactory bulb of mice. Biol Trace Elem Res 142: 683–692. 10.1007/s12011-010-8791-1 20680508

[52] Traystman RJ, Kirsch JR, Koehler RC (1991) Oxygen radical mechanisms of brain injury following ischemia and reperfusion. J Appl Physiol 71: 1185–1195. 1757340 10.1152/jappl.1991.71.4.1185

[53] Andersen JK (2004) Oxidative stress in neurodegeneration: cause or consequence? Nat Med 10 Suppl: S18–25. 15298006 10.1038/nrn1434

[54] Yadwad VB, Kallapur VL, Basalingappa S (1990) Inhibition of gill Na^+^- K^+^-atpase activity in dragonfly larva, *Pantala flavesens*, by endosulfan. Bull Environ Contam Toxicol 44: 585–589. 2157509 10.1007/BF01700880

[55] Samuel PM, Roy S, Jaiswal KA, Rao JV (1998) Differential effects of organometallic tin compounds on Na+/K+-ATPase activity. J Appl Toxicol 18: 383–386. 9804439 10.1002/(sici)1099-1263(1998090)18:5<383::aid-jat523>3.0.co;2-0

[56] Elsabbagh HS, Moussa SZ, El-tawil OS (2002) Neurotoxicologic sequelae of tributyltin intoxication in rats. Pharmacol Res 45: 201–206. 11884216 10.1006/phrs.2001.0909

[57] Hartl MG, Hutchinson S, Hawkins LE (2001) Sediment-associated tri-n-butyltin chloride and its effects on osmoregulation of freshwater-adapted 0-group European flounder, *Platichthys flesus* (L.). Aquat Toxicol 55: 125–136. 11595303 10.1016/s0166-445x(01)00184-9

[58] Oruc EO, Uner N, Tamer L (2002) Comparison of Na^+^-K^+^-ATPase activities and malondialdehyde contents in liver tissue for three fish species exposed to azinphosmethyl. Bull Environ Contam Toxicol 69: 271–277. 12107705 10.1007/s00128-002-0057-y

[59] Beliaeff B, Burgeot T (2002) Integrated biomarker response: A useful tool for ecological risk assessment. Environmental Toxicology and Chemistry 21: 1316–1322. 12069320

[60] Kim WK, Lee SK, Jung J (2010) Integrated assessment of biomarker responses in common carp (*Cyprinus carpio*) exposed to perfluorinated organic compounds. J Hazard Mater 180: 395–400. 10.1016/j.jhazmat.2010.04.044 20452121

[61] Li ZH, Velisek J, Zlabek V, Grabic R, Machova J, Kolarova J, et al (2011) Chronic toxicity of verapamil on juvenile rainbow trout (*Oncorhynchus mykiss*): Effects on morphological indices, hematological parameters and antioxidant responses. J Hazard Mater 185: 870–880. 10.1016/j.jhazmat.2010.09.102 20970250

[62] Feng M, Qu R, Li Y, Wei Z, Wang Z (2014) Biochemical biomarkers in liver and gill tissues of freshwater fish *Carassius auratus* following in vivo exposure to hexabromobenzene. Environ Toxicol 29: 1460–1470. 10.1002/tox.21876 23804377

